# Loss of Corticostriatal Mu-Opioid Receptors in α-Synuclein Transgenic Mouse Brains

**DOI:** 10.3390/life12010063

**Published:** 2022-01-03

**Authors:** Jessica Grigoletto, Meir Schechter, Ronit Sharon

**Affiliations:** Department of Biochemistry and Molecular Biology, IMRIC, Hadassah Medical School, The Hebrew University, Ein Kerem, Jerusalem 9112001, Israel; jessica.grigoletto@sns.it (J.G.); meir.schechter@mail.huji.ac.il (M.S.)

**Keywords:** α-Synuclein, mu-opioid receptors (MOR), white matter tracts (WMTs), corticostriatal glutamatergic axons

## Abstract

Ultrastructural, neurochemical, and molecular alterations within the striatum are associated with the onset and progression of Parkinson’s disease (PD). In PD, the dopamine-containing neurons in the substantia nigra pars compacta (SNc) degenerate and reduce dopamine-containing innervations to the striatum. The loss of striatal dopamine is associated with enhanced corticostriatal glutamatergic plasticity at the early stages of PD. However, with disease progression, the glutamatergic corticostriatal white matter tracts (WMTs) also degenerate. We analyzed the levels of Mu opioid receptors (MORs) in the corticostriatal WMTs, as a function of α-Synuclein (α-Syn) toxicity in transgenic mouse brains. Our data show an age-dependent loss of MOR expression levels in the striatum and specifically, within the caudal striatal WMTs in α-Syn tg mouse brains. The loss of MOR expression is associated with degeneration of the myelinated axons that are localized within the corticostriatal WMTs. In brains affected with late stages of PD, we detect evidence confirming the degeneration of myelinated axons within the corticostriatal WMTs. We conclude that loss of corticostriatal MOR expression is associated with degeneration of corticostriatal WMT in α-Syn tg mice, modeling PD.

## 1. Introduction

Parkinson’s disease (PD) is primarily defined as a movement disorder, although the patients present also non-motor and behavioral symptoms [1]. The striatum is a major input nucleus of the basal ganglia that receives dopaminergic innervation from the substantia nigra pars compacta (SNc). An additional major input to the striatum is the glutamatergic input from cortical and thalamic regions [2,3]. Data show that loss of dopaminergic innervation to the dorsal striatum is associated with enhanced glutamatergic input to this region [3,4], suggesting that alterations in dopaminergic and glutamatergic inputs to the striatum underlie its complex dysfunction, including motor and non-motor dysfunction, during the course of the disease.

Striatal synaptic transmission is modulated, among other factors, by the opioid system [5]. The four subtypes of opioid receptors, mu, delta, kappa, and nociceptin/orphanin FQ receptor (MOR, DOR, KOR, and NOP, respectively) are involved in the regulation of dopamine functions in the brain [5,6,7]. Opioid transmission in the basal ganglia has been implicated in PD [7,8,9,10] and enhanced opioid transmission is suggested to play a compensatory role in the altered basal ganglia function following depletion of striatal dopamine [8,9]. Striatal MOR expression is reported to play a role in hedonic processing and reward function [11,12,13], and alterations in the availability of striatal MOR may underlie a subset of disease symptoms [5].

The striatum is a heterogeneous mosaic of two neurochemically, developmentally, and functionally distinct compartments, the striosomes and the matrix [14]. MORs are expressed in the different neuronal populations and subregions of the striatum [13,15,16,17,18] including on the presynaptic, glutamatergic terminals of cortical neurons that are part of the corticostriatal white matter tracts (WMTs) [17,19], that are mostly assembled in striatal striosomes [20,21,22,23,24].

α-Synuclein (α-Syn) protein is implicated in the cytopathology and genetics of PD [25]. In the brain, α-Syn pathology, in the form of Lewy bodies and neurites, is strongly associated with disease severity [26,27]. In previous studies [28,29], we systematically investigated biochemical and ultrastructural alterations in the striatum of α-Syn tg mouse and human brains, as a function of disease progression. Focusing on striatal glutamatergic WMTs, we detected evidence for specific degeneration, including myelin loss and accumulation of α-Syn pathology, side by side with evidence for increased density of thin-diameter axons. Based on the results, we suggested the occurrence of two seemingly opposing mechanisms taking place in parallel, within striatal WMTs, namely, axonal degeneration and axonal arborization [28,29].

In this study, we asked whether MOR levels within the striatal glutamatergic tracts are altered in association with the changes occurring in the striatum of α-Syn tg mouse brains. Specifically, do MOR levels correlate with the degeneration of myelinated axons or with the increase in axonal arborization within striatal WMT? We report a progressive loss of MOR levels in striatal WMTs of α-Syn tg mouse brains along with the accumulation of α-Syn toxicity. The results highlight the importance of understanding the effects of α-Syn toxicity on corticostriatal opioid receptors in relevance to PD.

## 2. Materials and Methods

Human brains. Slides containing tissue sections of progressive PD (Braak stage 5–6) and age-matched control brains, containing caudate, putamen, and internal capsule (on one brain section) were supplied by the Multiple Sclerosis Society Tissue Bank, funded by the Multiple Sclerosis Society of Great Britain and Northern Ireland, registered charity 207495. Slides containing tissue sections of early PD cases (Unified stage IIa-IIb) and relevant control brains, containing the head of the caudate nucleus and internal capsule were provided by the Banner Sun Health Research Institute, Sun City, AZ, USA. The approval for the use of human tissue material was obtained from The Peer Review Panel of the Parkinson’s UK Brain Bank and the Brain Donation Program at Sun Health Research Institute. 

Mice. The human PrP-A53T α-Syn tg mouse line [30] was cross-bred with α-Syn-/- C57BL/6JOlaHsd mice (Harlan Laboratories, Jerusalem, Israel [31]) to delete endogenous mouse α-Syn; and then bred to achieve homozygosity of the human A53T α-Syn transgene. α-Syn-/- C57BL/6JOlaHsd genotype was used as control mice [31]. The PrP-A53T α-Syn tg model was shown in previous studies to develop motor disabilities and to accumulate α-Syn pathology in an age-dependent manner. That is, mice appear generally healthy and show no evidence of α-Syn pathology up to the age of 8–9 months [29,30,32]. However, at 12 months of age and older, the large majority of mice in the colony show signs of motor disabilities accompanied by pathogenic accumulations of α-Syn in the central nervous system. The number of sick mice grows with age and the oldest mice in the colony are ~15 months old. 

Thy-1 hWT α-Syn mice [33,34] were obtained from Prof. Eliezer Masliah (UCSD, San Diego, CA, USA). Control mice were non-transgenic littermates. The Thy-1 hWT α-Syn mice show early signs of learning and motor disabilities at 2–4 months of age, which worsen at 8–10 months of age [34,35]. α-Syn pathology for the Thy-1 hWT α-Syn mice was demonstrated at 8–12 months of age [29,34].

All animal welfare and experimental protocols were approved by the Committee for the Ethics of Animal Experiments of the Hebrew University of Jerusalem NIH approval # OPRR-A01-5011 (Permit number: MD-16-14826-3). 

Luxol Fast Blue staining. Paraffin-embedded mouse brain sections were processed as described previously [29]. Images were acquired using a Nikon Ti Eclipse motorized inverted microscope with DIC, phase epi-fluorescence optics, and a Perfect Focus System (PFS). Equipped with a Nikon DS-Fi1 color CCD camera and NIS-Elements image acquisition software.

Cell fractionation and Western blotting. Tissue punches containing the dorsal striatum or substantia nigra were removed from mouse brains as previously described [28]. Tissues were homogenized [28] and subjected to differential centrifugation to obtain a crude synaptosomal fraction (P10, pellet obtained post 200× *g* for 10 min), a plasma membrane-enriched fraction (P25, pellet obtained post 8000× *g* for 15 min), and a corresponding soluble fraction (S25, supernatant obtained post 8000× *g* for 15 min). Protein samples (30 μg) were loaded on a 12% sodium dodecyl sulfate-polyacrylamide gel electrophoresis (SDS-PAGE) and processed for Western blotting as described in [29]. Blots were immunoreacted with a polyclonal rabbit anti MOR ab (1:5000 Millipore, Rosh-Ha’ayin, Israel) or polyclonal rabbit anti-Calbindin-D-28-K (CB-28, 1:5000, Sigma, Rehovot, Israel). The immunoreactive signals were quantified using UN-SCAN-IT GEL 3.1 software (Silk Scientific, Orem, UT, USA). The signal obtained for MOR in P10 and P25 membrane fractions were normalized to the levels detected for Na+/K+/ATPase protein (1:10,000, Sigma, Rehovot, Israel), used as a loading control. The immunoreactive signal obtained for CB-28 (1:5000, C2724, Sigma-Aldrich, Israel) in the soluble fraction was normalized to immunoreactive signal obtained with anti Actin ab (1:5000, Sigma-Aldrich, Israel).

Immunohistochemistry (IHC). Paraffin-embedded, coronal mouse brain sections (6  μM) were processed for immunostaining as described previously [28,29]. Briefly, for the detection of NF-200 immunoreactivity, sections were treated for antigen retrieval at 96 °C for 15 min, in 10 mM citrate buffer, pH 6.0, using a pressure cooker (DC2012, Biocare Medical, Concord, CA, USA). Sections were let cool at RT × 20 min. Slides were washed in PBS and blocked in 10% normal goat serum (NGS, Jackson Laboratories, Bar Harbor, ME, USA) in 0.1M Tris-HCL pH 7.6 containing 0.3% Triton-100, for 2 h at RT. Slides were then incubated with the polyclonal rabbit anti-NF-200 antibody (1:200, Sigma-Aldrich, Rehovot, Israel) at 4C0 overnight. The secondary antibody was goat-anti rabbit Texas Red (1:80, Jackson Laboratories, ME, USA). For Myelin Basic Protein (MBP) staining, brain sections were immunoreacted with monoclonal rat anti-MBP (1:200, Serotec) simultaneously with the secondary donkey- anti-rat Alexa Fluor 647- conjugated (1:100, Jackson Laboratories, ME, USA) [29]. For detection of MOR immunoreactivity, antigen retrieval was at 80 °C for 10 min in 10 mM citrate buffer, pH 6.0. Slides were then incubated with the polyclonal rabbit anti-MOR (1:1000, AB 5511, Millipore, Rosh-Ha’ayin, Israel) at 4 °C overnight. Slides were washed and reacted with the secondary antibody goat-anti rabbit Cy2-conjugated (1:100, Jackson Laboratories, ME, USA) for 1 h at room temperature. Sections were washed and mounted (Sigma Aldrich, Rehovot, Israel). For the detection of CD-28K immunoreactivity, pressure cooker antigen retrieval was set at 60 °C for 10 min, in 10 mM citrate buffer, pH 6.0. Sections were incubated with the polyclonal rabbit anti-CD-28K antibody (1:3000, Sigma-Aldrich, Rehovot, Israel) at 4 °C overnight. Slides were then washed and reacted with the secondary antibody, goat-anti rabbit Cy2-conjugated (1:100, Jackson Laboratories, ME, USA) for 1 h at RT. Sections were washed and mounted in a mounting medium (Sigma Aldrich, Rehovot, Israel). Fluorescence images were acquired using a Zeiss LSM 710 Axio Observer confocal Z1 laser scanning microscope. All images were taken using the same settings. The specific signal inside WMTs was quantified per area and normalized to the signal outside of WMTs in the same section. The background was determined in a consecutive section, stained with the secondary ab, and subtracted. The field of view was 400  ×  400 μm. Two-dimensional images measuring 384  ×  384 µm and 10 µm per pixel optical resolution were created. Quantification was performed based on 6–10 non-overlapping images (from the two hemispheres) per brain. Image series were analyzed with Image pro plus 6.3 program (Media Cybernetics, Bethesda, MD, USA), using a size-based threshold. Fields were chosen randomly following the scan at a lower magnification. Images were taken and analyzed blindly to tissue classifying information.

Slides containing human brain sections of control or PD donors were processed for IHC as previously described [28]. Briefly, for antigen retrieval, slides were incubated in a pressure cooker at 110 °C for 15 min in 10 mM citrate buffer, pH 6.0. Blocking was in CAS-block solution (Thermo-Fisher, Rehovot, Israel) containing 0.3% Triton-100, for 10 min at RT. Slides were then reacted with both a monoclonal rabbit anti-SMI-32 antibody (1:1000, Covance Inc., Princeton, NJ, USA) and the polyclonal anti-MBP antibody (1:200, Sigma-Aldrich, Rehovot, Israel) at RT for 2 h. Slides were then washed and simultaneously reacted for 1 h at RT, with the host-corresponding secondary antibodies. The immunoreactive signal was captured by a confocal Zeiss LSM 710 Axio Observer. Z1 laser scanning microscope. In each experiment, exciting laser, intensity, background levels, photo multiplier tube (PMT) gain, contrast, and electronic zoom size were maintained at the same level. For each antibody, the background was subtracted (determined by a negative control consisting of a secondary antibody alone). The zoom of each picture was obtained by choosing the plane with the greatest fluorescent signal. 

Real-time quantitative polymerase chain reaction (q-PCR). Total RNA was extracted using TRIzol reagent (Invitrogen, Carlsbad, CA, USA), from tissue punches containing the striatum or substantia nigra from α-Syn and age-matched control mice. RNA quality and concentrations were determined by measuring the 260/230 nm ratio using a Nanodrop Spectrophotometer. 1000 μg RNA were reverse transcribed into cDNA by using the high capacity cDNA, reverse transcription Kit (Applied Biosystems, Foster City, CA USA). Primer pairs were designed to exon-exon boundaries by primer-BLAST software and used at the concentration of 0.25 μM. For the quantification of MOR and D1 expression, the RNA sample was pretreated with DNase solution (1 μg per μL; RQ1 RNase-Free DNase, Promega Corporation, Madison, USA) to eliminate DNA contaminations, as the set of primers that were used may also amplify a DNA template. Quantitative RNA levels were detected by the Applied Biosystems Step One Software v2.2.2 with SYBR Green Master Mix (Applied Biosystems Foster City, CA USA). The expression levels of 18S were used as a reference gene to normalize the data. The experiment was repeated 3 times for each gene and group. Primer’s sequences are listed in Table 1.

Statistical analysis. Comparisons between the groups were performed by *t*-test (Prism 7). Bonferroni correction was applied for multiple comparisons. All data were presented as mean ± SD.

## 3. Results

### 3.1. Loss of MOR Immunoreactivity within Corticostriatal WMTs of A53T α-Syn tg Mouse Brains

**Figure 1:**
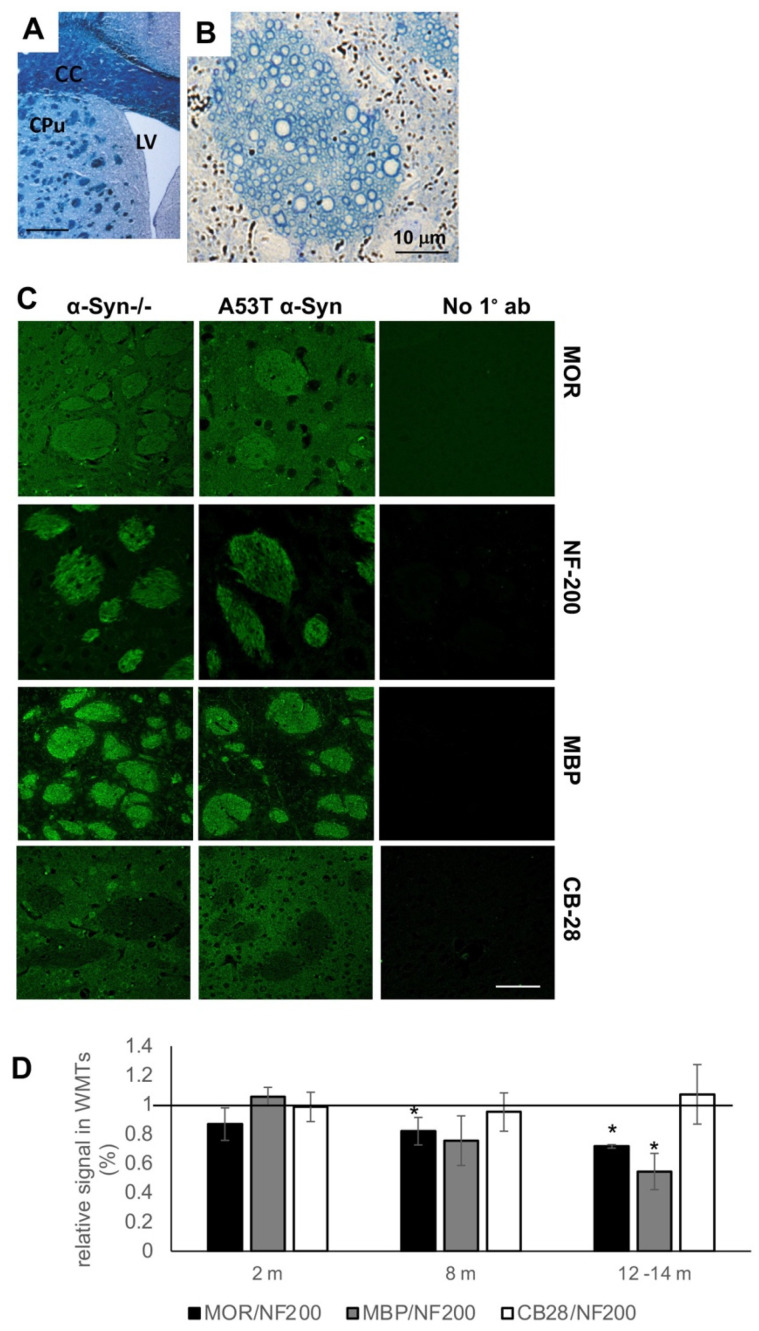
Lower MOR levels in corticostriatal WMT of A53T α-Syn tg mouse brains. (**A**). A lower magnification of a coronal mouse brain section, stained with Luxol Fast blue, showing the striatum. CC, corpus callosum; LV, lateral ventricle; CPu, caudate putamen. WMTs are shown as dark spots over the lightly stained striatal grey matter in the CPu. Bar = 0.5 mm. (**B**). A semi-thick A53T α-Syn mouse brain section (1 μm), stained with methylene blue, showing cross-sectioned axons in a WMT. Bar = 10 μm. (**C**). Paraffin-embedded coronal sections (6 μm) of α-Syn-/- (C57BL/6JOlaHsd) and A53T α-Syn mouse brains at 2 months of age, containing the dorsal striatum (representative images). Sections immunoreacted either with anti-MOR, anti-NF-200, anti-MBP, or anti calbindin 28 (CB-28) antibodies. The consecutive section of the α-Syn-/- brain were stained with the corresponding secondary antibody as a control (no 1°). Bar = 50 μm. (**D**). The relative WMTs immunoreactive signal of MOR or MBP normalized to the signal obtained for NF-200. Total CB-28 signal in striatal matrix normalized to NF200 signal. Mean ± SD of A53T mice at 2, 8, and 12 months of age. The horizontal bar represents the corresponding age-matched control mice, set at 100%. *n* = 4–5 brains for each genotype and each age group; 3–6 fields per brain; *t*-test with Bonferroni correction, * *p* < 0.017.

Striatal MOR levels were assessed by quantitative immunohistochemistry (IHC) in paraffin-embedded sections of A53T α-Syn tg and α-Syn-/- control mouse brains (*n* = 4–5 brains/genotype and age group). The tissue block was set to contain the dorsal/caudal striatum, to capture the glutamatergic efferents that enter the striatum through striatal WMTs [2]. Systematic quantification of MOR signal was carried out at three age groups representing stages of symptoms/pathology severity in this mouse model. Specifically, two months of age, representing young and healthy mice with no α-Syn-pathology; eight months, representing pre-symptomatic mice and 12–14 months, representing symptomatic mice with an apparent accumulation of α-Syn pathology [29,30]. MOR signal, detected within WMTs (Figure 1B,C) was normalized to the immunoreactive signal obtained for NF-200, which was found to be stable between 2–12 months of age [28] (Figure 1C,D).

Setting the normalized MOR signal in the control α-Syn-/- brains at 100% for each of the age groups tested, we detected 86.7 ± 10.9% at two months; a significantly lower 81.9 ± 9.3% at eight months and a significantly lower 71.6 ± 1.1% at 12–14 months old A53T α-Syn tg mouse brains (Figure 1D). Mean ± SD of *n* = 4–5 brains for each genotype and each age group (3–6 fields per brain; *t*-test, with Bonferroni correction, *p* < 0.017). Suggesting an age-dependent loss in MOR signal in the WMTs, in this mouse model. Starting as a tendency for a lower signal at the young age, before the appearance of symptoms, and reaching statistical significance at the older ages alongside with accumulation of evidence for α-Syn pathology [29,30]. 

In accord with our previous reports [28,29], the results also show an age-dependent loss of MBP signal, localized to striatal WMTs. That is, setting the signal ratio for MBP/NF-200 in control mice for each of the age groups at 100% (represented by the horizontal line in Figure 1D), we detected a similar signal ratio for the A53T α-Syn tg mice at two months. However, significant ~75% and ~55% lower signal ratios were detected at 8 and 12–14 months of age, respectively (t-test with Bonferroni correction, *p* < 0.017). We next immunoreacted the tissue sections with anti-calbindin-28 (CB-28) ab, as a marker for the striatal matrix. Importantly, no differences in the immunoreactivity of CB-28 were detected between A53T α-Syn and control mouse brains throughout the tested age groups. Together, the loss of striatal MOR signal correlates with axonal degeneration, represented by the loss of MBP signal within WMTs. 

### 3.2. Loss of MOR Protein Expression Levels in the Striatum of Thy-1 hWT α-Syn Mouse Brains

**Figure 2:**
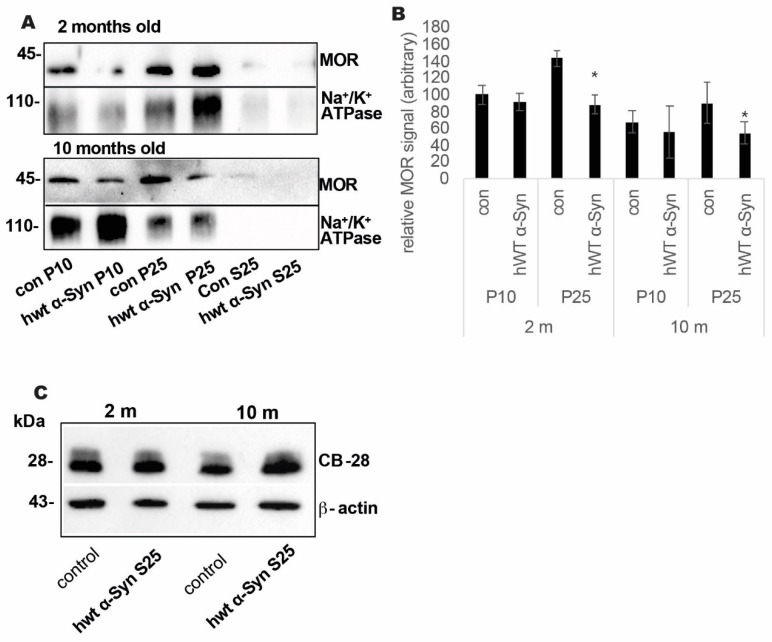
MOR levels are lower in the striatum of Thy-1 WT α-Syn tg mouse brains. (**A**). Samples of membrane fractions P10 or P25, or supernatant S25 (30 μg protein) obtained from tissue punches containing the striatum of Thy-1 α-Syn and control mouse brains at 2 and 10 months of age analyzed by Western blotting and immunoreacted with anti-MOR or anti-Na+/K+ ATPase antibodies. The original western blot images are provided in the Supplementary material Figure S1 (mouse brain samples at 2 months of age) and Figure S2 (mouse brain samples at 10 months of age). (**B**). Graph showing quantitation of blots obtained in (**A**) mean ± SD of *n* = 4 mice. *, <0.05, *t*-test. (**C**). Western blot as in (**A**), the soluble S25 fraction immunoreacted with anti-CB-28 or anti β-actin antibodies. The original western blot images are provided in the Supplementary material Figure S3 (left side shows mouse brain samples at 2 months of age; the right side shows mouse brain samples at 10 months of age).

The specific loss in MOR signal was next confirmed in the striatum of a second mouse model for PD, the Thy-1 hWT α-Syn mice (Figure 2A,B). Tissue punches containing the striatum at 2 and 10 months of age, representing early and progressive symptoms (respectively) for this mouse model [33,36], were homogenized immediately after dissection. A sequential centrifugation protocol resulted in biochemical fractionation of crude synaptosomes fractions (P10), a plasma membrane-enriched (P25), and a soluble fraction (S25) [37]. Analyzing these fractions by quantitative Western blotting, we detected MOR signals in both membrane fractions, P10 and P25. Only traces of MOR immunoreactivity were detected in the soluble S25 fraction. MOR signal in the P25 fraction from striata of Thy-1 hWT α-Syn mice was significantly lower than the signal detected in the age-matched control mice. That is, the signal detected in the P25 fraction obtained from Thy-1 hWT α-Syn at 2 or 10 months of age was calculated as ~60% of the signal detected in the age-matched control mice (*n* = 4–5 mice, in each group, analyzed in 2–3 blots *p* < 0.05, *t*-test). Analyzing the soluble S25 fractions for CB-28 protein immunoreactivity by Western blotting, we found no differences between the α-Syn and control samples (*n* = 4 mice/group analyzed in 2–3 blots *p* < 0.05, *t*-test) at both age groups tested (Figure 2C).

### 3.3. Loss of MOR Signals Occur at the mRNA Level

**Figure 3:**
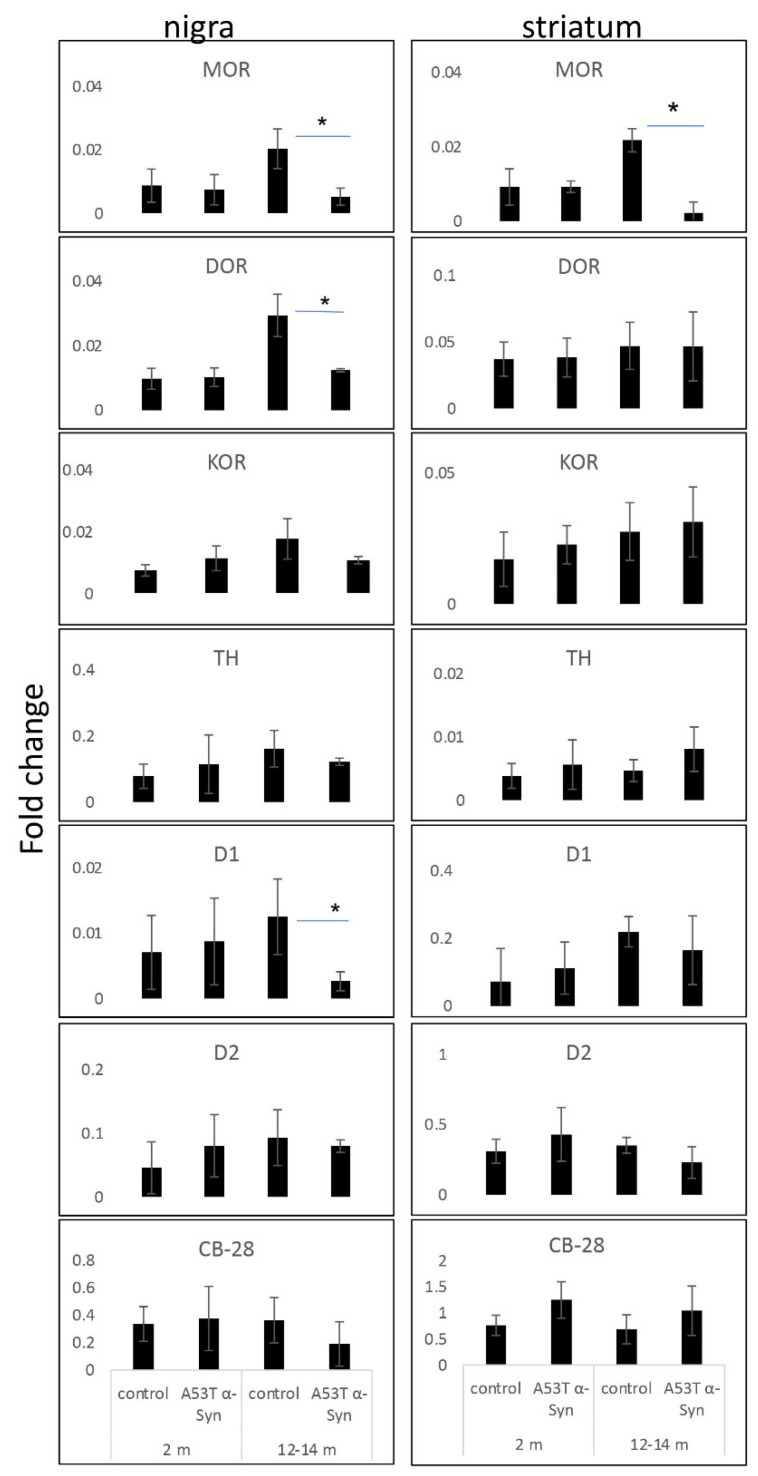
Lower mRNA levels of MOR in striatum and substantia nigra of A53T α-Syn tg mouse brains. Graphs showing qPCR values, detected following RNA extraction from tissue punches containing the striatum or SNc of A53T and age-matched control mouse brains, at 2 or 12–14 months of age (*n* = 4–5 mouse brains in each genotype and age group, tested in triplicates). The detected mRNA levels are normalized to the levels of 18S in the same sample. *, *p* < 0.007, *t*-test with Bonferroni correction for multiple comparisons.

A systematic gene expression analysis at the mRNA levels was performed for opioid receptors (MOR, DOR, and KOR); dopamine receptors D1 and D2; CB-28; and tyrosine hydroxylase (TH) by qPCR. RNA was extracted from tissue punches containing the striatum or nigra, from A53T α-Syn and control mouse brains at 2 or 12–14 months of age. The results show significantly lower mRNA levels for MOR in the striatum and nigra of the old A53T α-Syn tg mice. In contrast, DOR mRNA levels were not altered in the striatum of old A53T α-Syn mice, yet, were found significantly lower in the nigra. The levels of D1 dopamine receptors were lower in the nigra of aged A53T α-Syn mouse brains. In contrast, the D2 receptor, which is enriched in the striatal matrix, was not altered [14]. In line with previous reports of this mouse model [30], TH levels were not altered between the mouse genotypes. These results support the occurrence of age-dependent degenerative mechanisms, taking place in the A53T α-Syn tg mouse brains, that are associated specifically with MOR expression levels, both in the striatum and the nigra (Figure 3).

### 3.4. Degeneration of Corticostriatal WMT in Human Brains with PD

**Figure 4:**
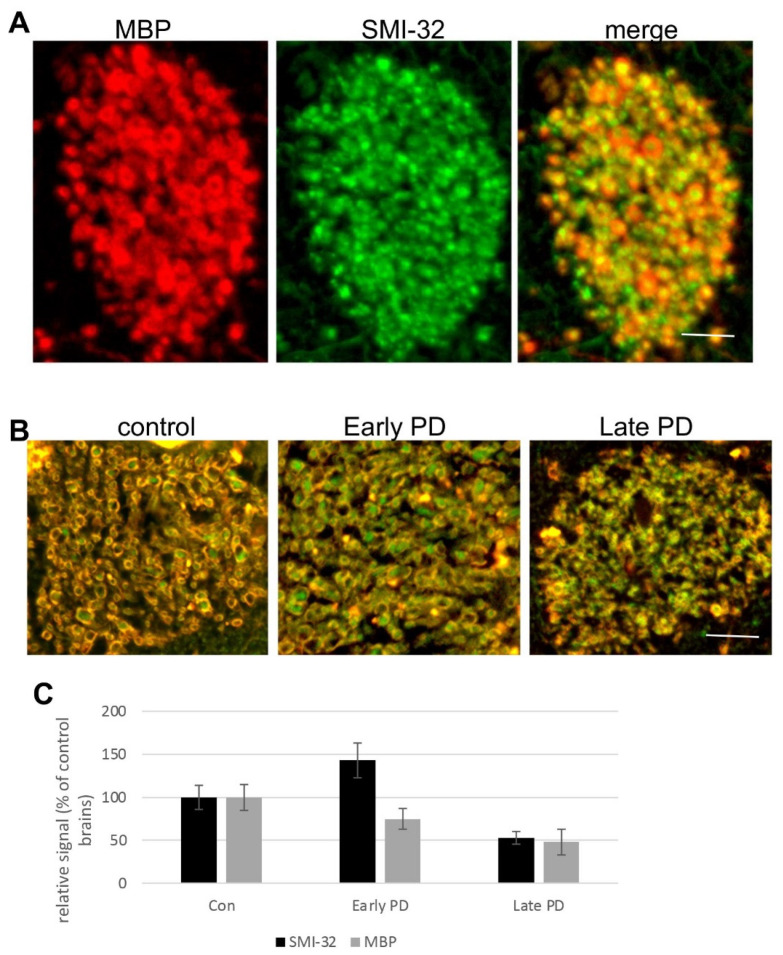
Axonal degeneration within caudal glutamatergic WMTs in progressive PD. (**A**). IHC of caudal WMTs in a human brain affected with early PD (male, 72 years, unified stage IIb). Brain section co-immunoreacted with anti-MBP and anti-SMI-32 antibodies. (**B**). IHC of a control human (male, 84 years); an early PD brain (as in **A**), and an advanced PD brain (male 75 years, Braak stage 5). Brain sections co-immunoreacted with anti-MBP and anti-SMI-32 antibodies. A representative brain is shown out of *n* = 3 in each group. Bar = 20 μm. (**C**). Quantitation of the signal as in (**B**) mean ± SD of *n* = 3 brains, 8–10 WMTs.

To follow up on our recent findings showing degeneration of the corticostriatal WMTs at advanced PD [28], we immunoreacted paraffin-embedded brain sections containing the caudate from early PD (unified stages IIa and IIb [27]; *n* = 3), advanced PD (Braak stages 5–6 [26], *n* = 3) and control (*n* = 3) cases with anti-SMI-32, a marker of glutamatergic corticostriatal connections [38] or anti-MBP antibodies. In line with the results obtained in the mouse [29] and human brains [28], we detected alterations within corticostriatal axons, localized at the caudate, during the course of the disease. Specifically, in early PD, axons within corticostriatal WMTs appeared intact with no signs of degeneration (Figure 4A). MBP immunoreactivity appears as a ring surrounding the axon and an SMI-32 signal is detected within the axon. However, at late stages of PD, the immunoreactive signals of MBP and SMI-32 are diffused, with no clear boundaries between the axons. Quantifying the immunoreactive signals obtained for MBP and SMI-32 within the corticostriatal WMTs indicated a loss of signal in the advanced stages of the disease (Figure 4B,C and [28]), supporting the occurrence of a degenerative mechanism within the glutamatergic corticostriatal WMTs. 

## 4. Discussion

This study examined alterations in levels of MOR expression, within the glutamatergic axon bundles that connect the cortex with the striatum. The protein and mRNA levels of MOR, detected within the corticostriatal WMTs or in tissue punches containing the striatum, were found lower in the α-Syn tg mouse models than their corresponding control mice. The results connect axonal degeneration within the corticostriatal WMTs with disease mechanisms in PD, and highlight the potential importance of MOR expression in corticostriatal WMTs, in the context of the non-motor symptoms of PD.

The study builds upon our previous reports showing that pathogenic alterations are taking place within the WMTs during the course of the disease. Specifically, we found evidence for an age-dependent accumulation of α-Syn expression and pathology within the cortico-striatal WMTs [29]. Interestingly, during the early stages of the disease, the pathogenic evidence coexisted with evidence for a higher density of axons within these cortico-striatal WMTs [28]. Supporting the occurrence of seemingly opposing mechanisms, including axonal damage side by side with axonal growth. Herein we report that alterations in MOR levels are associated with the degenerative process in the corticostriatal WMTs. 

The data point out an altered biochemical balance within the sub-compartments of the striatum of α-Syn tg mouse brains. That is, the levels of the striatal matrix marker CB-28, were unchanged whereas MOR-expressing glutamatergic WMT appeared to degenerate. The altered biochemical balance within striatal sub-compartments may contribute to the development of motor and non-motor symptoms that are associated with PD. For example, recent studies have demonstrated an involvement of these connections in cost-benefit decision-making and motivation mechanisms that are affected by chronic stress [39] and aging [40].

Previous studies have reported alterations in the levels of opioid receptor immunoreactivity in PD patients and animal models [41,42]. Lower opioid receptor binding was detected in the caudate of PD patients, and in the putamen and thalamus of dyskinetic PD patients compared to non-dyskinetic. Alterations in striatal MOR levels have been associated with L-DOPA-induced dyskinesia (LID), a common and disabling side effect of chronic use of L-DOPA in PD patients [5,41,42]. Individuals with LID exhibit abnormal levels of endogenous opioids [10,43,44]. However, inconsistent data obtained from preclinical and clinical studies have led to conflicting concepts that opioids represent either a cause of LID or a compensatory mechanism. Moreover, both MORs antagonism and agonism are being studied for their anti-dyskinetic properties [45,46,47]. Data obtained in models of LID [48] indicate increased sensitivity of MOR in the basal ganglia in the dyskinetic states [49]. We demonstrate alterations in expression levels of MOR, yet, the degree of MOR sensitivity or activity was not determined in this study. It is plausible that alterations in MOR sensitivity may accommodate the alterations in MOR expression levels. 

Studies in animal models suggest that the endogenous opioid system has an important role in controlling appetite in animal models [50,51,52,53]. Low MOR levels were detected in the dorsal striatum of patients affected with schizophrenia [12] and in long-term opiate drug users [54,55]. In contrast, high MOR levels in the ventral striatum are associated with alcohol dependence [56,57]; and higher MOR availability in the anterior cingulate and frontal cortex are associated with cocaine dependence [58]. These studies highlight the involvement of striatal MOR levels in behavioral abnormalities, and the potential implications for patients with PD.

Striatal mRNA levels show loss of MOR expression, yet DOR levels were unaffected in the striatum of α-Syn tg brains. In contrast, the mRNA levels of MOR’s and DOR’s detected in the SN, were lower in the old α-Syn tg compared with the control mouse brains. Interestingly, the A53T α-Syn tg mouse line does not recapitulate the degeneration of dopaminergic axons in the striatum nor degeneration of neuronal cell bodies in SN, which characterizes the disease [28,30]. Thus, it is not clear why the mRNA levels of MOR and DOR, detected in the nigra of α-Syn tg mice, are lower than those detected in the control brains. The loss of striatal MOR in α-Syn tg mouse brains may demonstrate a primary response to α-Syn toxicity rather than a secondary response to axonal degeneration in the striatum. With relevance to DOR, data support a protective role for DOR in models of PD. Pharmacological activation of DOR was shown to attenuate the toxic effects induced by treatments with MPTP, hypoxia, or α-Syn overexpression. In addition, DOR activation was suggested to inhibit α-Syn expression and aggregation [59,60]. Therefore, DOR activation may potentially present a therapeutic target for the treatment of PD.

## Figures and Tables

**Table 1:** Specific primers for real-time-PCR assay.

Gene	Prime Sequence (5′->3′)	Pre-Treatment Condition
18S	Forward: 5′-GCCAGAACCTGGCTGTACTT-3′ Reverse: 5′-GAGCGAGTGATCACCATCAT-3′	
MOR	Forward: 5′-GCCTTAGCCACTAGCACG-3′ Reverse: 5′-AACATTACGGGCAGACCA-3′	DNase *
DOR	Forward: 5′-ATCGTCCGGTACACCAAATTGA-3′ Reverse: 5′-GTACTTGGCGCTCTGGAAGG-3′	
KOR	Forward: 5′-GTTTGTCATCATCCGATACACGAA-3′ Reverse: 5′-GCATAGTGGTAGTAACCAAAGCATCT-3′	
CD-28K	Forward: 5′-CGCTGACGGAAGTGGTTACC -3′ Reverse: 5′-TTCCGGTGATAGCTCCAATCC-3′	
D1	Forward: 5′-AACTGTATGGTGCCCTTCTGTGG -3′ Reverse: 5′-CAGCCCCGTTGTTGTTGATG-3′	DNase *
D2	Forward: 5′CATCAGCATCGACAGGTACACA-3′ Reverse: 5′-CAGTAACTCGGCGCTTGGA-3′	
TH	Forward: 5′-AAATGCTGTTCTCAACCTG-3′ Reverse: 5′-GCTTCAAATGTCTCAAACAC-3′	

* DNAse treatment for sets of primers that amplify a region within one axon.

## Data Availability

All data generated or analyzed during this study are included in this published article.

## Supplementary Materials

The following supporting information can be downloaded at: https://www.mdpi.com/article/10.3390/life12010063/s1, Figure S1: Original blot immunoreacted with anti MOR or anti Na+/K+ ATPase antibodies, corresponding to Figure 2A; Figure S2: Original blot immunoreacted with anti MOR or anti Na+/K+ ATPase antibodies, corresponding to Figure 2A; Figure S3: Original blot immunoreacted with anti CB-28 or anti-actin antibodies, corresponding to Figure 2C.
