# Supplementary material for: Loss of Corticostriatal Mu-Opioid Receptors in α-Synuclein Transgenic Mouse Brains
Source: Life (Basel). 2022 Jan 3;12(1):63. doi: 10.3390/life12010063 (PMC8781165; doi:10.3390/life12010063)

Figure S1 Original blot immunoreacted with anti MOR or anti Na+/K+ ATPase antibodies, corresponding to Fig. 2A

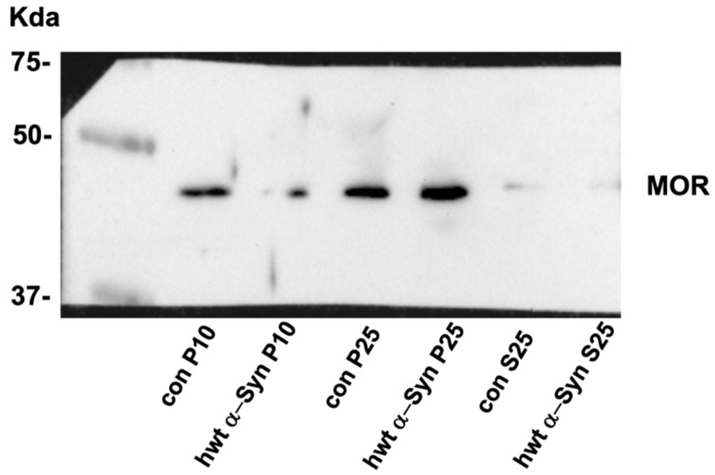

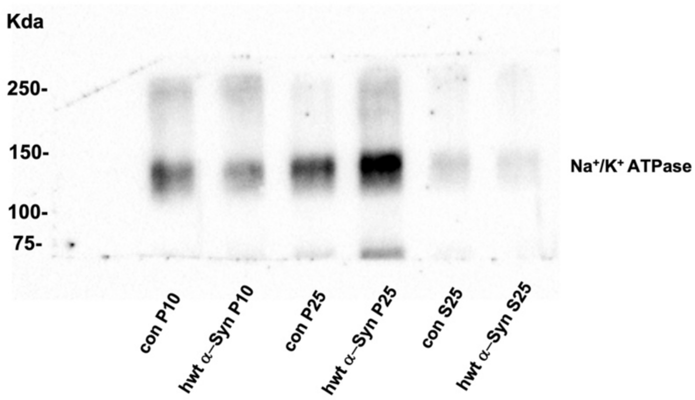

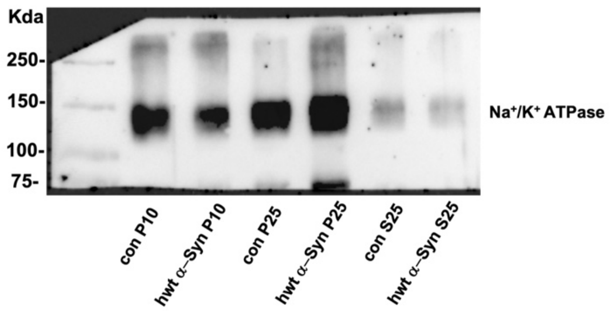

Figure S2 Original blot immunoreacted with anti MOR or anti Na+/K+ ATPase antibodies, corresponding to Fig. 2A

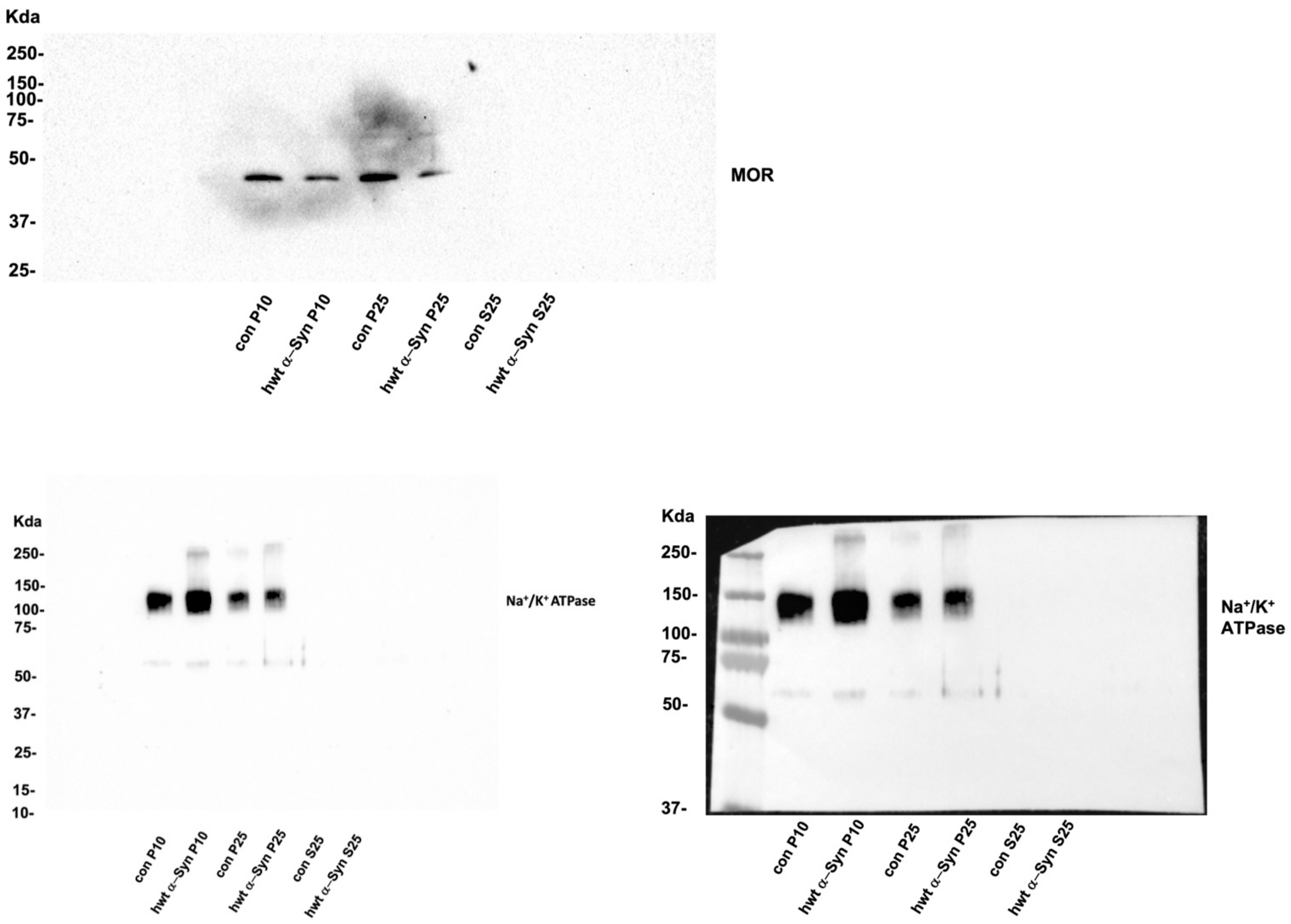

Figure S3 Original blot immunoreacted with anti CB-28 or anti β-actin antibodies, corresponding to Fig. 2C

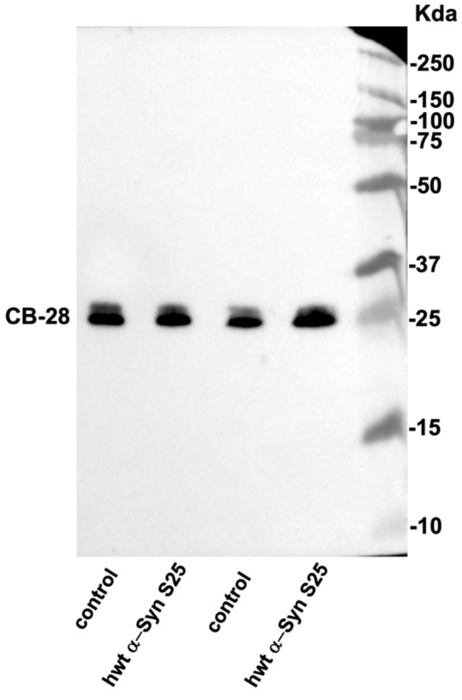

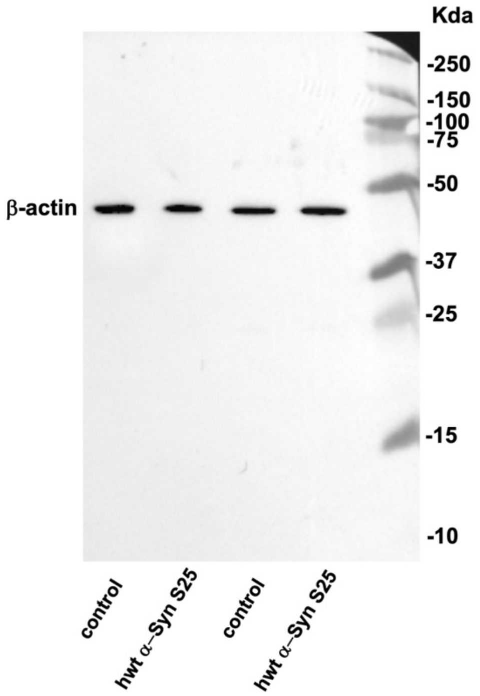

Supplement: Supplementary file 1 [file life-12-00063-s001.zip › life-1490141-supplementary.pdf]
